# Graph hierarchy: a novel framework to analyse hierarchical structures in complex networks

**DOI:** 10.1038/s41598-021-93161-4

**Published:** 2021-07-06

**Authors:** Giannis Moutsinas, Choudhry Shuaib, Weisi Guo, Stephen Jarvis

**Affiliations:** 1 School of Computing, Electronics and Mathematics, Coventry University, Coventry, UK; 2 Department of Computer Science, University of Warwick, Coventry, UK; 3 Centre for Autonomous and Cyberphysical Systems, Cranfield University, Cranfield, UK; 4 College of Engineering and Physical Sciences, University of Birmingham, Birmingham, UK

**Keywords:** Complex networks, Applied mathematics

## Abstract

Trophic coherence, a measure of a graph’s hierarchical organisation, has been shown to be linked to a graph’s structural and dynamical aspects such as cyclicity, stability and normality. Trophic levels of vertices can reveal their functional properties, partition and rank the vertices accordingly. Trophic levels and hence trophic coherence can only be defined on graphs with basal vertices, i.e. vertices with zero in-degree. Consequently, trophic analysis of graphs had been restricted until now. In this paper we introduce a hierarchical framework which can be defined on any simple graph. Within this general framework, we develop several metrics: hierarchical levels, a generalisation of the notion of trophic levels, influence centrality, a measure of a vertex’s ability to influence dynamics, and democracy coefficient, a measure of overall feedback in the system. We discuss how our generalisation relates to previous attempts and what new insights are illuminated on the topological and dynamical aspects of graphs. Finally, we show how the hierarchical structure of a network relates to the incidence rate in a SIS epidemic model and the economic insights we can gain through it.

## Introduction

Patient zero is the start of an epidemic that spreads through a city. A rumour spreads like wildfire amongst a group of friends. An accident happens on the road and the associated disturbance spreads congestion throughout the road network in the vicinity of the incident. These are just a small number of examples of real life processes involving the directed flow of some quantity, whether it be information or physical, across a graph structure. Graphs are omnipresent and they constitute many of the complex systems that underlie much of our infrastructure and social interactions as well as ecological and biological systems that control and regulate life. Since the turn of the millennium there has been an explosion of research in network science^1,2^. Understanding how signals or processes percolate through a graph and what role topology and structure play, has been a key research aim^3^.

Hierarchical structure is pervasive across complex networks with examples spanning from neuroscience^4^, economics^5^, social organisations^6^, urban systems^7^, communications^8^, pharmaceuticals^9^ and biology^10^, particularly metabolic^11^ and gene networks^12^. Previous work, such as^13–17^ and^18^ attempt to further advance the study of hierarchical structures in complex graphs from various perspectives. These approaches typically cast hierarchy into a dichotomy; hierarchy in terms of a modular organisation known as nested hierarchy, an instance of community structure, or alternatively, a directed flow hierarchy. Our framework covers both the order and flow hierarchy approaches. Our work directly generalises the trophic approach and makes its topological connections explicit.

Graphs play a significant role in ecology where graph tools are used to understand the complex ecosystems and food webs that are present in our environment^19,20^. Ecological networks are directed graphs representing biological interactions and exhibit a natural trophic structure. Researchers have defined the notion of trophic levels to illustrate the hierarchical nature of these graphs^21^. The trophic incoherence parameter was introduced as a measure of how neatly a graph can be partitioned into discrete trophic levels. Research has shown that trophic incoherence is a proxy for the stability of a food web^22^ and found that the lack of cycles in a graph is inherently linked with the trophic coherence of a graph^23^. These ideas have been used to analyse the spread of infections on a graph^24^, and to assess robustness and resilience of rail networks^25^. This illustrates a link between the stability and dynamics of graph processes, and the underlying structure of the graph.

Trophic analysis can only be conducted for graphs with basal vertices, i.e. vertices with no in-edges. In this paper we propose a framework that generalises trophic analysis to any graph. We introduce two new centrality measures and two new graph coefficients:

- *Hierarchical levels* is a generalisation of the notion of trophic levels and describes each vertex’s rank with respect to “energy or “information” flow.
- *Influence centrality* is a measure of a vertex’s ability to influence the long term state of the graph.
- The *democracy coefficient* measures the feedback that is present in the graph.
- The *hierarchical incoherence parameter* is a straightforward generalisation of trophic incoherence.

Vertices with high influence centrality have different functions in different contexts. In ecology, these are the basal species at the bottom of the food web; in epidemiology, they form the outbreak zone; and in morning transportation, the commuter towns. This is intimately tied with the study of the controllability of complex systems^26^.The democracy coefficient is intimately related to the source subgraphs of the graph, which are subgraphs that are not influenced by the rest of the graph. We explore the effect that the democracy coefficient and the hierarchical incoherence parameter have on the spread of an infection by modelling a contagion dynamics.

## Preliminaries

In this article we follow the convention of flow networks and we will call a vertex with no in-neighbours a *source*. Such vertices in the context of food webs are called basal. Similarly, a vertex with no out-neighbours will be called a *sink*.

We will consider weighted simple directed graphs and we will use *G* to denote them. If a graph is undirected, it can be turned into a directed graph by replacing each undirected edge by a pair of directed edges. We will denote by $G^T$ the transpose graph of *G*, i.e. the graph we get if we reverse the direction of all edges in *G*. We will denote by ${{\,\mathrm{So}\,}}(G)$ and ${{\,\mathrm{Si}\,}}(G)$ the set of all source and sink vertices, respectively. Moreover, if *H* is a subgraph of *G*, we will denote by $G\setminus H$ the graph that we get by removing from *G* all vertices in *H* with all the edges that are adjacent to them. We will call the “graph” $G\setminus G$ the *empty graph*.

We will shift our point of view from energy flow to information flow and we consider the following dynamics on a weighted graph. We assign a colour to each vertex. Then, at each time step a vertex chooses at random a vertex between itself and its in-neighbours with a probability proportional to the weighted in-degree. Its own weight is always 1. Then all vertices update their colour to the colour of their chosen vertex simultaneously. We will call this *forward influence dynamics*. In this dynamics, source vertices are important because they stay in their original colour forever. We define *backward influence dynamics* to be the same process on the transpose graph, i.e. the colour of a vertex is updated by considering the out-neighbours instead of the in-neighbours. Similarly to the forward influence dynamics, a sink vertex will remain in its original colour for ever.

### Definition 2.1

A graph *G* is called *simply forward influenced* if ${{\,\mathrm{So}\,}}(G)$ and $G\setminus {{\,\mathrm{So}\,}}(G)$ are not empty and for any $v\in G \setminus {{\,\mathrm{So}\,}}(G)$ there exists $u\in {{\,\mathrm{So}\,}}(G)$ such that there is a directed path from *u* to *v*. Similarly, *G* will be called *simply backward influenced* if its transpose graph $G^T$ is *simply forward influenced*.

Food webs are typically simply forward influenced. We prove in the Supplemental Material (SM) that trophic levels can be defined on a graph if and only if it is simply forward influenced. Sources in a graph do not have to be single vertices, they can also be subgraphs. This leads us to the following definition.

### Definition 2.2

Let *G* be a weakly connected graph and $\Gamma$ a subgraph of *G*. A subgraph $\Gamma$ is called a source subgraph of *G* if there is no edge from $G\setminus \Gamma$ to $\Gamma$. A source subgraph $\Gamma$ is a *minimal source subgraph* of *G* if the only subgraph of $\Gamma$ that is a source subgraph of *G* is $\Gamma$ itself. A subgraph $\Gamma$ is a *minimal sink subgraph* if it is a minimal source subgraph for the transpose graph, $G^T$.

**Figure 1:**
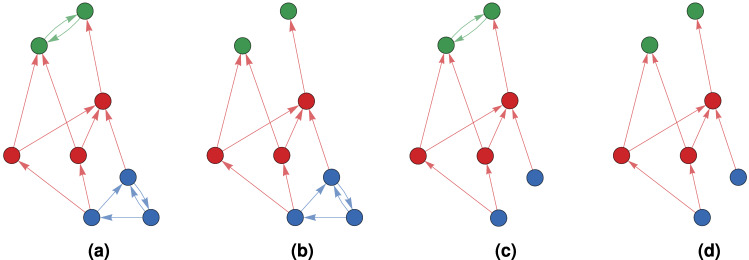
An example of a hierarchically decomposable graph. (**a**) The original graph. Its source subgraph is marked blue and its sink subgraph is marked green. If we apply the forward influence dynamics on the graph, then in finite time all vertices will become blue. On the other hand if we apply the backward influence dynamics, then in finite time all vertices will become green. In both cases, red vertices do not affect the asymptotic state. (**b**) The simply forward influenced subgraph. (**c**) The simply backward influenced subgraph. (**d**) The core subgraph.

For example in Fig. 1a the subgraph that consists of the blue and the red vertices is a source subgraph, however it is not minimal. The blue subgraph is a minimal source subgraph, since if we make it any smaller it stops being a source subgraph. We show later that a minimal source subgraph is strongly connected.

### Definition 2.3

For a weakly connected graph *G*, let $\Gamma _1$, $\dots$, $\Gamma _l$ be its minimal source subgraphs and let $\Delta _1$, $\dots$, $\Delta _l$ be its minimal sink subgraphs. The subgraph $F_f$ (resp. $F_b$) that we get if we remove all edges that belong to $\Gamma _i$’s (resp. $\Delta _i$’s) from *G* and delete all isolated vertices is called the *simply forward* (resp. *backward*) *influenced subgraph* of *G*. We call the set $\{\Gamma _1$, $\dots$, $\Gamma _l$, $F_f\}$ (resp. $\{ \Delta _1$, $\dots$, $\Delta _l$, $F_b\}$) the *forward* (resp. *backward*) *hierarchical decomposition* of *G*. The subgraph *C* that we get if we remove all edges that belong to $\Gamma _i$’s and $\Delta _i$’s from *G* and delete all isolated vertices is called *core subgraph* of *G* and the set $\{\Gamma _1$, $\dots$, $\Gamma _l$, *C*, $\Delta _1$, $\dots$, $\Delta _l\}$ is called the *hierarchical decomposition* of *G*.

If the hierarchical decomposition of *G* is non-trivial, i.e. $\Gamma _1=\Delta _1=G$, we call *G* hierarchically decomposable.

If a graph *G* is strongly connected, its hierarchical decomposition is trivial. We prove in SM that a graph is not hierarchically decomposable if and only if it is strongly connected. Notice that if *G* is hierarchically decomposable, then $G^T$ is also hierarchically decomposable. An example of a hierarchically decomposable graph, its maximal simply influenced subgraphs and its core subgraph can be seen in Fig. 1.

The hierarchical decomposition of a graph can be compared to the bow-tie structure of graphs^27^. The input comprises of the source subgraphs and the output comprises of the sink subgraph. Finally, the core is comprised of the vertices that cannot reach the input and cannot be reached by the output. The difference is that in our case we do not impose the restriction that the core has to be strongly connected.

### Definition 2.4

Let *G* be a hierarchically decomposable graph and $H_1$, $\dots$, $H_l$ be its minimal source (resp. sink) subgraphs. A vertex $v\in G$ is called a *forward* (resp. *backward*) *influencer* if $v\in \cup _i H_i$ and *v* is called *forward* (resp. *backward*) *influenced* if $v\in G\setminus \cup _i H_i$.

### Notation

We will use *A* to represent the weighted adjacency matrix of a simple positively weighted graph and we will denote its entries by $a_{ij}$. If there is no directed edge between *i* and *j* then we have $a_{ij}=0$ and if there is a directed edge from *i* to *j*, then $a_{ij}$ is positive and represents the edge’s weight.

We define $d_i=\sum _{j}a_{ji}$ to be the weighted in-degree of vertex *i* and $d=(d_1,\dots ,d_n)$ to be the weighted in-degree vector. The weighted in-degree Laplacian of a graph is defined to be the matrix $L=\text {diag}(d)-A$. For notational convenience we define $M=L^\mathsf {T}$, where $L^\mathsf {T}$ is the transpose of *L*. Similarly we define $\delta$ to be the weighted out-degree vector and the out-degree Laplacian of a graph is the matrix $\Lambda = \text {diag}(\delta ) - A$.

## Hierarchical levels and hierarchical differences

In this section we introduce several, local and global, graph metrics. The notions of hierarchical levels and influence centrality are local metrics that can be used as vertex centrality/ranking measures. Forward hierarchical levels are a direct generalisation of trophic levels. We discuss the relation between trophic levels and hierarchical levels further in SM. Hierarchical levels can be computed on any positively weighted simple directed graph. The notions of democracy coefficient and hierarchical incoherence are global metrics that characterise the graph. The notions of influence centrality and democracy coefficient have explicit connections with the topology of the graph. In both cases we define two versions of the metrics, forward and backward, capturing the different notions of control and dependence. The backward metrics are equivalent to the forward versions applied to the transposed graph.

### Hierarchical levels

We now define the notions of hierarchical levels. Forward hierarchical levels are defined through the matrix *M* and backward hierarchical levels are defined through the matrix $\Lambda$. We will call the difference of forward and backward hierarchical levels simply hierarchical levels.

#### Definition 3.1

Let *G* be a directed graph, *d* and $\delta$ be its in-degree and out-degree vectors respectively, *L* and $\Lambda$ are the in-degree and out-degree Laplacian matrices respectively and $M=L^\mathsf {T}$. The vector of *forward hierarchical levels*, *g*, is

$$\begin{aligned} g:=\underset{x\in {\mathcal {T}}}{{{\,\mathrm{arg\,min}\,}}}\Vert x\Vert _2 \text {, where } \mathcal {T}:=\underset{x\in \mathbb {R}^n}{{{\,\mathrm{arg\,min}\,}}}\Vert M x-d\Vert _2. \end{aligned}$$

Similarly the vector of *backward hierarchical levels*, $\gamma$, is

$$\begin{aligned} \gamma :=\underset{x\in {\mathcal {S}}}{{{\,\mathrm{arg\,min}\,}}}\Vert x\Vert _2 \text {, where } {\mathcal {S}}:=\underset{x\in \mathbb {R}^n}{{{\,\mathrm{arg\,min}\,}}}\Vert \Lambda x-\delta \Vert _2. \end{aligned}$$

Finally, we define the vector of *hierarchical levels* of *G* to be $h=\frac{1}{2}(g-\gamma )$.

We note that by this definition we obtain $g=M^+ d$ and $\gamma = \Lambda ^+\delta$, where $M^+$ denotes the Moore-Penrose inverse of the matrix *M*, see^28^. Intuitively, forward hierarchical levels grade the vertices based on their distance from source subgraphs. Backward hierarchical levels rank the vertices based on their distance from sink subgraphs. They provide the perspectives of control and dependence respectively. Hierarchical levels take into account both perspectives and can be used to layout a graph in a way that reveals its overall hierarchical structure.

The computation of the varying hierarchical levels boils down to minimising a sum of squares, for which we have reliable and efficient algorithms available^29^. By definition it is easy to see that the forward (resp. backward) hierarchical levels of a graph *G* are equal to the backward (resp. forward hierarchical) levels of $G^T$, respectively. This means that the vector of hierarchical levels of $G^T$ is $-h$, where *h* is the vector of hierarchical levels of *G*.

**Figure 2:**
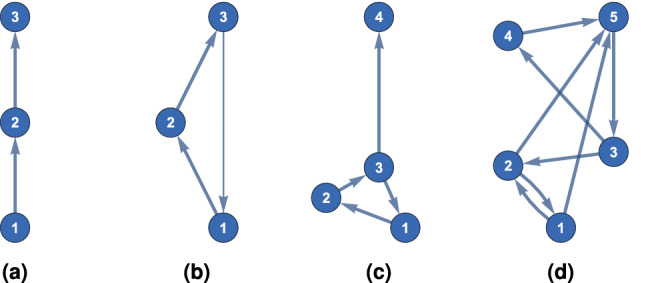
Examples of hierarchical layout of graphs: The *y* coordinate of a vertex corresponds to its hierarchical level. In (b) the edge $3\rightarrow 1$ has smaller weight than the other two edges.

Some simple examples are illustrated in Fig. 2. The *y* coordinate of a vertex corresponds to its hierarchical level. In Fig. 2a a directed 3-chain is shown. The forward and backward hierarchical levels of the vertices are $(-1,0,1)$ and $(1,0,-1)$, respectively. The hierarchical levels are $(-1,0,1)$, which agrees with our intuition.

In the case of a directed 3-cycle, because of its symmetry we expect that all vertices will have the same hierarchical level 0. We break the symmetry of the 3-cycle in two ways. In Fig. 2b we set the weight of the edge $3\rightarrow 1$ to be 1/2 and the rest of the weights equal to 1. This is halfway between a 3-chain and a 3-cycle. Intuitively, we expect that in this case vertex 1 should be lower than the rest. We find that the forward and backward hierarchical levels of the vertices are $(-0.5,0,0.5)$ and $(0.5,0,-0.5)$ respectively, which implies that the hierarchical levels are $(-0.5,0,0.5)$.

The other way we break the 3-cycle’s symmetry is by adding another vertex, Fig. 2c. In this case the forward hierarchical differences of the 3-cycle do not change, only the hierarchical levels change. The forward hierarchical levels are $(-0.25,-0.25,-0.25,0.75)$. However when we look at the backward hierarchical levels, we see that the vertices of the 3-cycle are not equivalent. The backward hierarchical levels are $(2.25, 1.25, 0.25, -3.75)$, so the hierarchical levels are $(-1.25, -0.75, -0.25, 2.25)$.

The final example, Fig. 2d, is a strongly connected graph. Intuitively we expect that in a strongly connected graph, vertices with low in-degree and high out-degree will tend to have low hierarchical levels. However, the hierarchical level of a vertex is not a local property and it depends also on the closed paths that start and finish on the vertex. The forward hierarchical levels are $(-0.783, -0.0333, -0.117, 0.3, 0.633)$ and the backward hierarchical levels are $(0.725, 0.607, 0.336, -0.868, -0.8)$. The hierarchical levels of this graph are approximately $(-0.754, -0.32, -0.226, 0.584, 0.717)$.

Additionally, because our measure can be applied to strongly connected graphs, this means it is applicable to undirected graphs but with a caveat. In the case of undirected graphs the adjacency matrix is symmetric and so the forward and backward hierarchical levels would be equal to each other. Consequently, the hierarchical levels would be zero. For an undirected graph we need to utilise only the forward hierarchical levels. Initial exploration shows that in the undirected scenario, where the density of the graph is greatest, the forward (resp. backward) hierarchical levels of vertices is greatest, compartmentalising the graph into core, gateway and peripheral divisions. Thus offering a potential method of detection of core-periphery structure^30^.

### Hierarchical differences

Hierarchical levels assign a numerical label to each vertex. We can use their differences to assign a numerical label to each edge. We will see that the weighted mean of hierarchical differences is an important metric. This leads us to the following definition.

#### Definition 3.2

The *forward*, respectively *backward, democracy coefficients* of a positively weighted simple directed graph *G* are defined to be

$$\begin{aligned} \eta _f(G) := 1-{{\,\mathrm{Mean}\,}}({{\,\mathrm{FHD}\,}}(G)) \;\;\;\text { respectively }\;\;\; \eta _b(G) := 1-{{\,\mathrm{Mean}\,}}({{\,\mathrm{BHD}\,}}(G)), \end{aligned}$$

where ${{\,\mathrm{FHD}\,}}(G) = \{ g_j-g_i \, |\, a_{ij} >0,\; i,j\in G \}$, are the forward hierarchical differences and ${{\,\mathrm{BHD}\,}}(G) = \{ \gamma _i-\gamma _j \, |\, a_{ij} >0,\; i,j\in G \}$, are the backward hierarchical differences. The mean is taken with respect to the edge weights.

Intuitively the democracy coefficient is a measure of how much the influencers of a graph are being influenced themselves. A low democracy coefficient means that there is minimal feedback between the influencer vertices, the vertices driving the dynamics, and the influenced vertices. Similarly to the trophic incoherence parameter we define the hierarchical incoherence parameter.

#### Definition 3.3

The *forward*, respectively *backward, hierarchical incoherence parameters*, or just *forward (resp. backward) hierarchical incoherences*, of a directed graph *G* are defined to be

$$\begin{aligned} \rho _f(G)=\sqrt{{{\,\mathrm{Var}\,}}({{\,\mathrm{FHD}\,}}(G))} \;\;\;\text { respectively }\;\;\; \rho _b(G)=\sqrt{{{\,\mathrm{Var}\,}}({{\,\mathrm{BHD}\,}}(G))}, \end{aligned}$$

where the variance is computed with respect to the edge weights.

The democracy coefficient together with the hierarchical incoherence for a given graph, give an insight to its topology. For a graph, high democracy coefficient and low hierarchical incoherence means that all its vertices have approximately the same hierarchical level; the graph is influenced by a large percentage of its vertices. On the other hand, low democracy coefficient and low hierarchical incoherence means that there are distinct hierarchical levels; the graph is controlled by a small percentage of its vertices. A graph is maximally hierarchical if both the democracy coefficient and the hierarchical incoherence are 0. This implies the vertices can be grouped in “layers”. All vertices in a layer have the same hierarchical level and hierarchical levels of two layers differ by an integer. Moreover, there can only be edges from one preceding layer to the layer succeeding it. Figure 2a, a directed chain, illustrates a prime example. This compares more favourably against other measures which need to alter the graphs in order to quantify hierarchy^18^.

We refer back to Fig. 2 to give an intuitive feeling for how these global graph metrics characterise graph structures. In Fig. 2a both the forward and backward democracy coefficients and hierarchical incoherence parameters are 0. For Fig. 2b both the forward and backward democracy coefficients are 0.8 and hierarchical incoherence parameters are 0.6. In Fig. 2c the forward democracy coefficient is 0.75 and forward hierarchical incoherence parameter is 0.433. The backward democracy coefficient is 0 and backward hierarchical incoherence parameter is 2.12. Finally, in Fig. 2d the forward democracy coefficient is 0.729 and forward hierarchical incoherence parameter is 0.693. The backward democracy coefficient is 0.667 and backward hierarchical incoherence parameter is 0.885.

The democracy coefficient has more interesting connections with the topology of the graph. A useful property of the democracy coefficients is that if *G* is weakly connected, then we can calculate its forward (resp. backward) democracy coefficients by calculating only the hierarchical levels of its minimal source (resp. sink) subgraphs. This is stated and proven in SM.

#### Lemma 3.4

*For a weakly connected directed graph* *G*, $\eta _f(G)\ge 0$ *and* $\eta _b(G)\ge 0$. *Moreover*, *G* *is a simply forward influenced graph if and only if* $\eta _f(G)=0$. *Similarly*, *G* *is a simply backward influenced graph if and only if *$\eta _b(G)=0$.

#### Lemma 3.5

*If* *G* *is a balanced graph, i.e. for any vertex its in-degree equals its out-degree, then* $\eta _f(G)=\eta _b(G)=1$.

By consequence of the above definition, the democracy coefficient of an undirected graph is 1. We conjecture that the democracy coefficient has also the following properties.

#### Conjecture 3.6

*Let* *G* *be a weakly connected directed graph. Then the following are true*:

- $$\eta _f(G) \le 1$$ *and* $$\eta _b(G) \le 1.$$
- $\eta _f(G)=\eta _b(G)=1$ *if and only if the graph is balanced*.

If the above upper bounds are correct, then the total weight of edges in the forward (resp. backward) influenced subgraph bounds the forward (resp. backward) democracy coefficient and the converse. We would get $\eta (G)\le 1-m/n$ and $m\le n(1-\eta (G))$, where *m* is the sum of weights of all edges in the simply forward (resp. backward) influenced subgraph and *n* is the total weight of all edges in *G*. Furthermore, the hierarchical differences can be used to define a new graph centrality measure.

#### Definition 3.7

The *forward* respectively *(backward) influence centrality* of vertex *j* of a positively weighted simple directed graph *G* are respectively defined to be

$$\begin{aligned} \eta _f(G,j)= 1 - {{\,\mathrm{Mean}\,}}({{\,\mathrm{FHD}\,}}(G,j)) \;\;\;\text { respectively }\;\;\; \eta _b(G,j)= 1 - {{\,\mathrm{Mean}\,}}({{\,\mathrm{BHD}\,}}(G,j)), \end{aligned}$$

where ${{\,\mathrm{FHD}\,}}(G,j) = \{ g_j-g_i \, |\, a_{ij} > 0,\; i\in G \}$, ${{\,\mathrm{BHD}\,}}(G,j) = \{ \gamma _i-\gamma _j \, |\, a_{ij} > 0,\; i\in G \}$ and the mean was taken with respect to the edge weights with the convention that the mean of the empty set is 0.

Influence centrality can be used to characterise a vertex as an influencer or not. If the influence centrality of a vertex is positive, then the vertex is an influencer.

#### Lemma 3.8

*Let* *G* *be a positively weighted, weakly connected, directed graph and* $i\in G$. *Then for any vertex* *i*, $\eta _f(G,i)\ge 0$, $\eta _b(G,i)\ge 0$ *and* *i* *is forward (resp. backward) influenced if and only if* $\eta _f(G,i) = 0$ *(resp*. $\eta _b(G,i) = 0$).

Moreover, there exist $i\in G$ such that $\eta _f(G,i)>0$ and $\eta _b(G,i)>0$, if and only if *G* is strongly connected.

This offers an explanation of the meaning of influence centrality. Alternatively, we can view it as the amount by which we need to change the degree vector of a graph in order to make *g* the satisfy the linear equation in its definition. More precisely, if *e* is the vector of forward influence centralities, then it is true that $Mg=d\circledast (\mathbf {1}-e)$, where $\circledast$ is the element-wise product. A similar relation holds for the backward influence centrality, see the SM for more details.

In strongly connected graphs, influence centralities can be related to the stationary distribution of a random walk.

#### Lemma 3.9

*Let* *G* *be a strongly connected graph*, $\Delta$ *be its weighted out-degree diagonal matrix,* $\epsilon$ *be the vector of backward influence centrality of* *G* *and let* $\pi$ *be the stationary distribution of a random walk on* *G*. *Then* $\pi =\Delta ^2\epsilon /\Vert \Delta ^2\epsilon \Vert _1$.

In the above lemma we assumed that at each step the random walker chooses randomly an out-edge with probability proportional to that edge’s weight. If the graph is weakly connected, then a random walker on it will eventually be trapped in a minimal sink subgraph. This means that we can apply Lemma 3.9 on the minimal sink subgraph and get the stationary distribution of the random walk there. Moreover, it is easy to see that all vertices with 0 backward influence centrality are transient states for the random walk. The relationship with random walks is further discussed in the SM.

This new graph centrality measure is able to determine the vertices with non-zero influence centrality and hence those that drive the asymptotic dynamics of all vertices in the graph. We note that the hierarchical level of influencer vertices differ depending on their position and their connection to the rest of graph. By further studying the profile of influence centralities and the democracy coefficient one could develop a control profile/signature for graphs and use it as a comparison of controllability across different graph structures. Ruths et al^31^ have answered this by using the degree distribution of a graph and degree properties of a vertex. We believe that influence centrality and hierarchical levels could produce a more accurate representation of the control properties of a complex graph.

**Figure 3:**
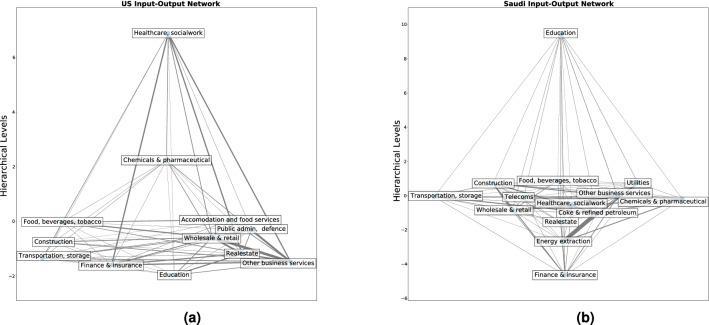
The relationships between the important economic sectors of the US and of Saudi Arabia represented as graphs.

**Figure 4:**
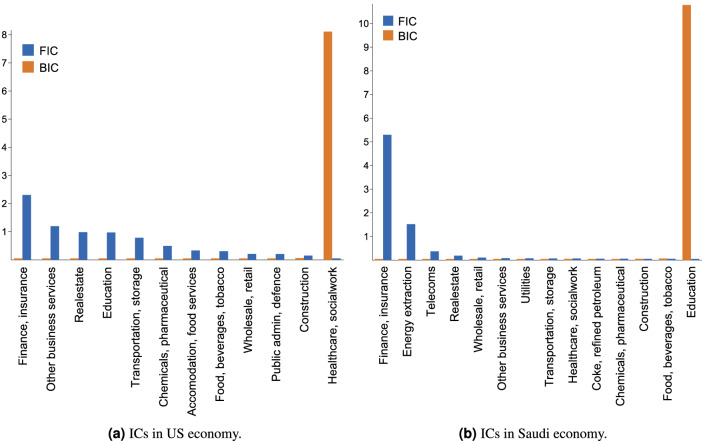
The influence centralities of the important economic sectors in the US and in Saudi Arabia.

### Illustration

We now illustrate an example of the utility of our framework in economics where the concepts of ’upstreamness’ and ’downstreamness’ of industries in production chains are used to study economic output. This is modelled by a complex directed graph of vertices which represent economic sectors and edges which represent the transfer of value between the sectors, in the form of goods and/or services^32^. The edges are weighted by the total monetary value of these transactions. This methodology was inspired by trophic analysis of food webs. In this case the trophic level of a vertex is called its *downstreamness* or its *output multiplier*. Hierarchical levels takes into account both the forward and backward ranking, thus incorporating the notions of demand and supply into its ranking. This is an interesting application for our methodology because the graphs are typically not simply forward influenced, since virtually every sector interacts with every other sector.

We have visualised the most significant sectors of the US and Saudi Arabia economies, Fig. 3. The choice of countries is due to the fact that their economies have significant structural differences. This data was constructed from the OECD Input-Output tables. The vertical position of each vertex corresponds to its hierarchical level. We have also computed the forward and backward influence centralities of these sectors, Fig. 4.

The forward influence centrality (FIC) of a vertex measures the extent in which the vertex functions as a source of the graph. We see that almost every sector of the US economy has positive FIC. The only exception is the Healthcare/Socialwork sector. In contrast only a handful of sectors of the Saudi Arabian economy have positive FIC. As a result, the forward democracy coefficient of the US graph is 0.502 and the forward democracy coefficient of the Saudi graph is 0.09. This means that in the US economy, there are more sectors which add value and that even though there exists a general direction to the flow of value, there is a lot of feedback. In contrast, in the Saudi economy a few sectors add value and most function as an intermediate, by transferring value between other sectors. This is also reflected in Fig. 3, where we see that the vertices in the US graph accumulate in the bottom of the figure, whereas in the Saudi graph accumulate in the middle.

It is interesting to note that in the US economy the vertex with the lowest hierarchical level is education, however the vertex with the highest FIC is Finance/Insurance. However, further investigation is needed before we can discuss the reasons for this discrepancy as well as the relative importance of these sectors in the economy. It is also interesting to note that the vertex with the lowest hierarchical level and the highest FIC in the Saudi graph is Finance/Insurance and not energy extraction as the authors expected. This raises interesting questions about the importance of the Finance/Insurance in energy extraction, however this question further investigation too.

The backward hierarchical structure of the two graphs is much more similar. Both have a single vertex that acts as a sink. That is Healthcare/Socialwork in the case of US and Education in the case of Saudi Arabia. The backward democracy coefficient for both graphs is virtually 0. This means that there is minimal feedback between the sink and the rest of the graph. This is also apparent in Fig. 4 where we see the BICs of all but one sectors in both graphs vanish. This raises an interesting question about the similarities between the function of the Healthcare/Socialwork sector in the US economy and the function of the Education sector in the Saudi economy, however this is beyond the scope of the current article.

We see that the Graph Hierarchy framework shines new light on exploring the links between economic sectors and their role in economic performance; it provides new information that needs to be studied further in determining which industries are crucial to driving economic performance.

### Related work

There has been prior research which attempted to tackle the hierarchical nature of complex graphs as mentioned in the introduction. Some attempts can treat only unweighted graphs^6,15^. Others define only a global hierarchical coefficient^33^. There are some approaches with a similar stratagem that have attempted to quantify hierarchy by generalising trophic analysis, see^34^ and^35^. These approaches however cannot differentiate vertices in the same strongly connected component and they do not have any explicit links to the topology of the graph. There is definite scope for further study and an initial direction to explore is shown in^36^. Additionally, random walks have been used to provide a hierarchical analysis of complex graphs, further strengthening the connection between random walk theory and Graph Hierarchy^37^.

The pseduoinverse of the Laplacian matrix has been used to calculate nodal spreading capacity^38^ and topological centrality^39^. A significant computational complexity advantage is present in our approach as the underlying computational algorithm does not calculate the pseudoinverse, but rather the product of the pseudoinverse with the degree vector. This makes the algorithm memory efficient as well as offering a significant time complexity advantage by utilising efficient convex optimisation algorithms. The time complexity depends on the sparsity of the graph and if the graph is sparse, like most real world graphs are, then complexity approaches *O*(*N*) in the number of vertices. The complexity in the worst case scenario occurs when the graph is dense and is $\sim O(N^3)$. This compares favourably with other approaches quantifying the hierarchical nature of graphs which provide less information, see^14,16^ and^18^. Additionally in the SM we compare it against popular centrality metrics for further context.

Additionally, graph study is typically classified into three levels:

1. *Microscopic* constitutes the study of individual vertices to understand their behaviour. Degree or centrality measures are typically used. Hierarchical level and influence centrality provide this level of granular analysis.
2. *Mesoscopic* concerns the study of groups or community structure. At this level it is interesting to study the interaction of vertices over short distances or classification of vertices. Hierarchical differences could provide this classification via a functional clustering algorithm. Vertices within an integer hierarchical difference of each other belong to the same functional module in the graph, simultaneously addressing the order hierarchy question.
3. *Macroscopic* classifies the global structure of the graph. Typically relevant parameters are average degree, average path length and average clustering coefficient. Hierarchical incoherence and democracy coefficient provide relevant parameters characterising the graph on the global scale.

Graph Hierarchy provides all this analysis within one coherent and comprehensive framework, a huge advantage over other approaches attempting to quantify hierarchy within complex graphs. Furthermore, within this construct we have laid the groundwork for a novel approach to controllability of complex graphs with the new metrics of influence centrality and democracy coefficient.

Graph Hierarchy generates an intriguing relationship with normality^40^. In the case of an undirected graph, the adjacency matrix is symmetric and hence no deviation from normality, but this is not true for a directed graph. There is clearly a strong correlation between trophic incoherence and the non-normality of the adjacency matrix^41^ and so the relationship with hierarchical incoherence requires further investigation. How these two impact the dynamics of a graph process and hence the stability requires further study. We note that the advantage of the hierarchical approach is that it has both a microscopic lens as well as a macroscopic one.

## Contagion dynamics

As an application, we look at contagion dynamics on directed graphs. We used a simple Susceptible-Infected-Susceptible epidemic model^42^. Following^24^, we define the probability that vertex *i* is infected at time $t+1$ to be

$$\begin{aligned} \mathbb P(i\text { is infected at time }t+1) = f_i(t)^a, \end{aligned}$$

where $f_i(t)$ is the fraction of *i*’s in-neighbours which are infected at time *t* and $\alpha$ is a positive parameter that controls the infection rate. The smaller $\alpha$ is, the easier it is for a vertex to be infected. We show that the forward hierarchical incoherence parameter has the same predicting ability as the trophic incoherence parameter on graphs whenever both can be defined, however the new generalisation can be used in more general settings.

### Graph generation

We generate graphs using a version of the *preferential preying model* (PPM), introduced in^22^, modified to not have source vertices. We call this model *non-source preferential preying model* (NSPPM). This modification creates graphs which are not simply forward influenced because they lack source vertices, but which are similar to PPM graphs. In SM we give the algorithms for generating both PPM and NSPPM graphs. Both models have a temperature parameter, *T*, which controls how hierarchical they are. Low temperature creates graphs with low hierarchical incoherence. High temperature creates graphs which have high hierarchical incoherence, that are similar to Erdös-Rényi graphs. In SM we also show that on PPM graphs the trophic incoherence parameter and the forward hierarchical incoherence parameter agree.

**Figure 5:**
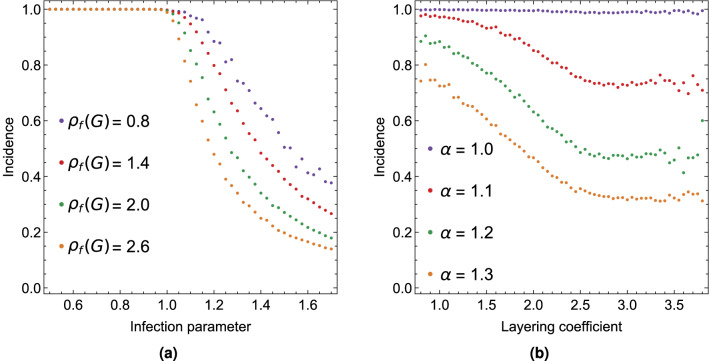
Scatter plot of average incidence values from Monte Carlo simulations of the infection spreading with varying hierarchical incoherence $\rho _f(G)$ and infection parameter $\alpha$. The average is taken over an interval of hierarchical incoherence values. (**a**) Incidence against $\alpha$ for different values of $\rho _f(G)$. (**b**) Incidence against $\rho _f(G)$ for different values of $\alpha$.

### Monte Carlo simulations

We ran Monte Carlo simulations using NSPPM graphs with 500 total vertices and 2500 edges with varying *T*. We start the simulation by infecting the 25 vertices with the lowest hierarchical levels and measure the incidence of the infection, i.e. the proportion of vertices that have been infected at least once. Then we run the simulation until either incidence becomes 1 or there is no infected vertex left or time step 1000 has been reached. The graph size was restricted by the complexity of the SIS model. The time needed for the computation of the graphs’ hierarchical structure was negligible, see SM for more information.

We observed that NSPPM graphs can be broken down into two categories based on the democracy coefficient. Graphs with democracy coefficient less than 20/2500 have a few small forward influencing subgraphs which start infected and stay infected forever. This means that depending on $\alpha$ either everything becomes infected or the algorithm times out. Graphs with democracy coefficient greater than 20/2500 tend to have bigger forward influencing subgraphs which do not stay infected. The two types of graphs exhibit different dynamical behaviour and we are able to identify this based only on the democracy coefficient. We discuss this in more detail in SM.

In Fig. 5 we show the average incidence for graphs with democracy coefficient 20/2500. Since we cannot choose values for the hierarchical incoherence, the average is taken over small intervals. We see that if $\alpha \le 1$ or smaller then the average incidence is almost 1, which means that every vertex becomes infected at least once. When $\alpha >1$, then incidence depends on the hierarchical incoherence of the graph. As expected, lower hierarchical incoherence corresponds to higher incidence. This means that we can use the information from the hierarchical structure of the graph and the parameter of the infection and predict the expected incidence without the need of simulations.

## Conclusion and future work

We have generalised the previous notions of trophic levels and coherence such that a trophic analysis can be applied to any simple graph, whether it be directed or undirected. The trophic approach has been previously applied to complex systems, particularly ecology, but with enforced restrictions that trophic analysis induces. Examples beyond the list given in the introduction, where our newly developed generalisation could advance previous work, include financial markets^43^ and water distribution networks^44^. This now enables some key questions concerning graph dynamics to be answered in a novel way: What is the resilience and robustness of a graph and how does it adapt under failure or attack? How does the topology of a graph affects its dynamics? How is the critical component of a graph determined when a flow is spreading across the structure and how does it rewire or redistribute flow under changing conditions?

Finally, we have developed open source Python and Julia packages, named GraphHierarchy, for researchers to easily apply these methods to a myriad of domains. We see this being extensively applicable as the graph abstraction is widespread in the analysis of complex systems. As such the potential of this approach to advance the study of the interplay between graph topology and dynamics is immense and leaves plenty of room for future work and collaboration.

## Supplementary Information

Supplementary Information.
