# Supplementary material for: Graph hierarchy: a novel framework to analyse hierarchical structures in complex networks
Source: Sci Rep. 2021 Jul 6;11:13943. doi: 10.1038/s41598-021-93161-4 (PMC8260706; doi:10.1038/s41598-021-93161-4)
Supplement: Supplementary file 1 — Supplementary Information. [file 41598_2021_93161_MOESM1_ESM.pdf]

Supplemental Material: Graph Hierarchy

Giannis Moutsinas[1], Choudhry Shuaib[2], Weisi Guo[3], and Stephen Jarvis[4]

[1]School of Computing, Electronics and Mathematics, Coventry University, Coventry, UK
[2]Department of Computer Science, University of Warwick, Coventry, UK
[3]Centre for Autonomous and Cyberphysical Systems, Cranfield University, Cranfield, UK
[4]College of Engineering and Physical Sciences, University of Birmingham, Birmingham, UK

The present document is the Supplemental Material of the article *Graph Hierarchy: A novel framework to analyse hierarchical structures in complex networks.*

Contents

1 Trophic Levels and Trophic Differences **2**
1.1 Trophic levels 2
1.2 Trophic differences 2
1.3 Hierarchical levels of simply forward influenced graphs 3

2 Connection with Random Walk **5**
2.1 Simply Influenced Graphs 5
2.2 Strongly Connected Graphs 5
2.3 Weakly Connected Graphs 6

3 Proofs **6**

4 Graph Generation **13**
4.1 Preferential Preying Model 13
4.2 Non-Source preferential preying model 14

5 Contagion Dynamics **16**

6 Time complexity **18**

1 Trophic Levels and Trophic Differences

The concept of trophic levels was introduced in [1] as a way to determine the hierarchy of species in a food chain. Primary producers, for example plants, have trophic level 1 and the trophic level of every other species is 1 plus the average trophic level of the species it eats. Interconnected food chains form what is called a food web. In a perfectly layered food web, all species have integer trophic levels and the difference between the trophic levels of the prey and the predator is 1. This rarely in practice happens and the notion of the trophic incoherence parameter was introduced as a way to measure how far a food web is from being perfectly layered.

1.1 Trophic levels

We represent a food web by a positively weighted, directed, simple graph. Typically, the direction of arrows indicate the flow of energy. We define the positive in-degree by $\tilde{d}_i = d_i$ if $d_i > 0$ or $\tilde{d}_i = 1$ if $d_i = 0$. We also define the positive in-degree vector $\tilde{d} = (\tilde{d}_1, \dots, \tilde{d}_n)$ and the positive in-degree Laplacian by $\tilde{L} = \tilde{D} - A$, where $\tilde{D} = \operatorname{diag}(\tilde{d})$. Finally, we define $\tilde{M} = \tilde{L}^\mathsf{T}$. Trophic levels are defined by the following linear equations:

$$\begin{aligned} s_i &= 1 + \frac{1}{d_i} \textstyle\sum_j a_{ji} s_j, && \text{if } d_i \neq 0, \\ s_i &= 1 && \text{if } d_i = 0. \end{aligned} \tag{1}$$

Using our notation we can write this system of equations in a compact form: $\tilde{M}s = \tilde{d}$. This leads to the following definition.

Definition 1.1. Let G be a simply forward influenced, positively weighted, simple graph. Then the vector of trophic levels on G is $s = \tilde{M}^{-1}\tilde{d}$.

The matrix $\tilde{M}$ is invertible if and only if the graph is simply forward influenced. An example of such graph can be seen in Figure 1.

1.2 Trophic differences

Trophic difference, i.e. the difference of trophic levels between two vertices connected by an edge, can be used to get a measure of how close a graph is to being perfectly layered. We define the trophic differences of a simply forward influenced graph G to be the set $\mathrm{TD}(G) = \{s_j - s_i \,|\, a_{ij} > 0\}$.

Lemma 1.2. Let G be a simply forward influenced, positively weighted, simple graph, then $\mathrm{Mean}(\mathrm{TD}(G)) = 1$, where the mean is taken with respect to edge weights.

We give the proof of this lemma in Section 3. Since the mean is always 1, the standard deviation of $\mathrm{TD}(G)$ can be a measure of the distance to a perfectly layered graph and is called the *trophic incoherence parameter* or just *trophic incoherence* of the graph. It is defined by

$$q(G) = \sqrt{\frac{\sum_{ij} (s_i - s_j)^2 \, a_{ij}}{\sum_{ij} a_{ij}} - 1}.$$

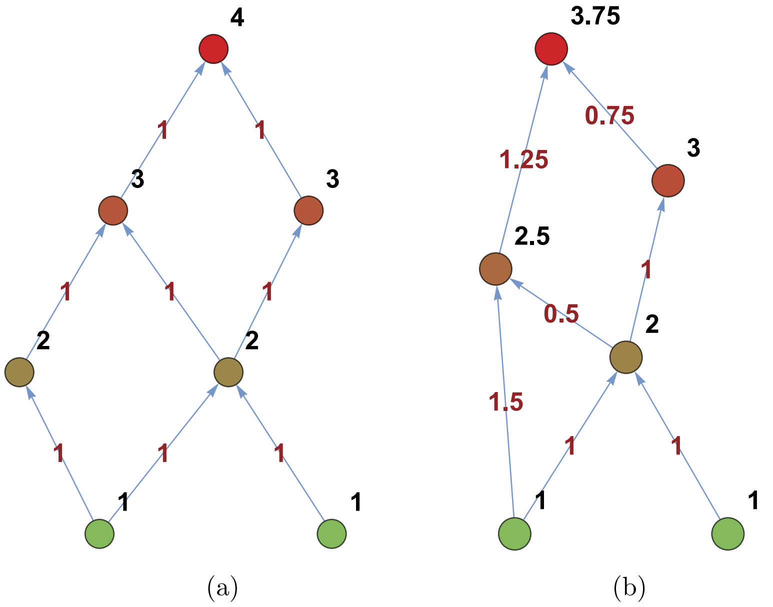

Figure 1: Two graphs representing two different food webs. Trophic levels are printed in black and trophic differences in red. (a) A totally coherent graph with integer trophic levels and trophic incoherence 0. (b) A less coherent graph with non-integer trophic levels and trophic incoherence 0.322.

1.3 Hierarchical levels of simply forward influenced graphs

In order to make the connection between trophic levels and forward hierarchical levels clear, we will discuss the case of simply forward influenced graphs more extensively. Let us consider linear system (1) and rewrite the first equation multiplied by d_i and using g instead of s as the unknown.

$$d_i g_i - \sum_j a_{ji} g_j = d_i. \tag{2}$$

Notice that if $d_i = 0$, the equation is trivially satisfied as it becomes $0 = 0$. Using our notation we rewrite equations (2) as

$$Mg = d. \tag{3}$$

Because M is a singular matrix, the above linear system does not have a unique solution. For a simply forward influenced graph the dimension of the kernel of M equals the number of source vertices. This means that we can get a unique solution by choosing arbitrary values of the trophic levels of the source vertices, see Lemma 3.1. We recover the original definition of trophic levels by setting the trophic levels of all source vertices to 1. However, using this viewpoint, we see that the choice of 1 is somewhat arbitrary and any other choice is equally valid. Instead of prescribing the trophic levels of source vertices we use Definition 3.1. In this case, because the linear system $Mx = d$ can be solved, we have $\mathcal{T} = \{x \in \mathbb{R}^n | Mx = d\}$. We find

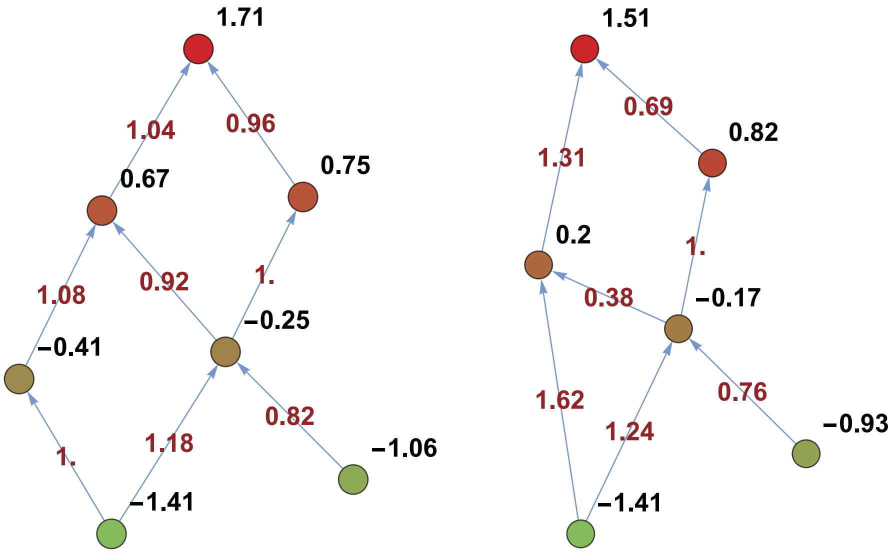

(a) (b)

Figure 2: Hierarchical levels and differences on the same graphs as in Figure 1. Forward hierarchical levels are printed in black and forward hierarchical differences in red. (a) The source vertices do not have the same forward hierarchical level and the hierarchical incoherence is 0.107. (b) A less coherent graph with a hierarchical incoherence of 0.423.

$$g = \arg\min_{x \in \mathcal{T}} \|x\|.$$

We will not discuss backward hierarchical levels here, as there is no corresponding notion in food webs.

We see in Figure 2 that the hierarchical levels of source vertices, which are typically called basal vertices in food webs, are typically not equal. This may seem strange for a food web, however it is worth noticing that the source vertex with the lowest hierarchical level is the root vertex for more food chains than the other source vertex. In general the length of the food chains also plays a role. This shows that the hierarchical levels have the added benefit of not treating all source vertices equally and from this we can deduce which basal species are more important in a food web.

Influence centrality Another way to view influence is as how much we need to "change" d in order to make the system $M\,g = d$ solvable. More precisely we have the following lemma.

Lemma 1.3. *Let G be a positively weighted, weakly connected graph, let d be its in-degree vector, let $M = \Lambda^T$, let g be its vector of forward hierarchical levels and let e be*

its vector of forward influence centrality of G. Then

$$Mg = d \circledast (\mathbf{1} - e) \tag{4}$$

where $\circledast$ denoted the element-wise product of vectors and $\mathbf{1}$ denotes the vector which has all its entries equal to 1.

This means that if we know e, then the system (4) can be solved. However, the solution is not unique. The freedom we have in solving this system is exactly the freedom of choosing the hierarchical level of one vertex in each source subgraph.

2 Connection with Random Walk

2.1 Simply Influenced Graphs

Unsurprisingly, the trophic levels of a graph have an explicit connection to random walk defined on the same graph. We consider an unweighted simply influenced graph, for example one of the two graphs shown in Figure 1. If we now consider a random walker, who picks an in-neighbour of the vertex and moves there, i.e. moves against the direction of edges. In order to distinguish this random walker from a typical one, we will call it a *random source seeker*[1].

Any vertex without in-edges, is going to be a trapping vertex for a random source seeker. Recall that in simply influenced graphs such vertices are called sources. Given that the graph is simply influenced, we know that any source seeker will eventually be trapped on a source vertex. Then it is natural to ask what is the average trapping time of a random source seeker that starts on a given vertex.

Clearly, if the source seeker starts on a source vertex, then the trapping time is 0. If the source seeker starts on any other vertex, then the average trapping time is the average trapping time of its in-neighbours plus 1.

When we compare this with the definition of trophic levels, we see that it is virtually the same bar the value at source vertices. This in practice means that the average trapping time for each vertex equals its trophic level minus 1, see [2].

When we consider the hierarchical levels on simply influenced graphs, the description does not change a lot. The only difference is the hierarchical level of the source vertices, now they do not have the same value. In fact the hierarchical level of a source vertex correlates with its probability of being the attracting vertex for a random source seeker. However, the nature of this correlation is non-trivial and requires further investigation.

2.2 Strongly Connected Graphs

Strongly connected graphs sit on the other side of the spectrum. In strongly connected graphs we cannot define trapping states in any meaningful way, so even the conceptual connection to trophic levels is completely lost. However, the connection with random walks does not disappear, it merely changes nature. We have the following result.

[1] Note that a random source seeker on a graph is the same as a random walker on the transpose graph.

Lemma 2.1. Let G be a positively weighted, strongly connected graph, let D be its in-degree and diagonal matrix, let e be its vector of forward influence centrality of G and let p be the stationary distribution of a random source seeker on G. Then

$$p = \frac{D^2 e}{\|D^2 e\|_1}.$$

Note that the above lemma is in practice Lemma 3.9 applies on G^T. The lemma gives an an explicit relation between the stationary distribution of a random source seeker and the forward influence centralities of a graph, however this relation is not symmetric in nature. If we know the forward influence centralities we can trivially compute the stationary distribution. However, knowing the stationary distribution, means that we still need to solve a linear system in order to compute the forward influence centralities. Equation (5) connects the stationary distribution to forward hierarchical levels.

2.3 Weakly Connected Graphs

In graphs that are neither simply influenced nor strongly connected, the relationship between a random source seeker and the hierarchical structure is a combination of both of the above. Any such graph can be decomposed to a trophic subgraph and a set of source subgraphs, see Definition 4.

The hierarchical level of a vertex of the simply influenced subgraph is equal to the average trapping time of its in-neighbours plus 1. In this case we consider the source seeker trapped once it enters a source subgraph. This means that the hierarchical levels of the simply influenced subgraph are uniquely and trivially determined by the hierarchical levels of the source subgraphs.

On the other hand, each source subgraph is strongly connected and can be considered on its own. The hierarchical differences of a source subgraph do not change if we consider it as a graph on its own right. Only the hierarchical levels change. This means that Lemma 2.1 holds on source subgraphs.

Similarly to source vertices the hierarchical levels of a source subgraph correlate with its probability of being the attracting subgraph for a random source seeker. As before the nature of this correlation is non-trivial and requires more investigation.

3 Proofs

In this section we provide the proofs of the lemmas in Section 3.2. The proofs are not written in the order that the lemmas appear in Section 3.2, but in the order they are used in other proofs, i.e. a lemma is used in a proof only if its proof was written before.

Lemma 3.1. Let G be a simply forward influenced graph with l source vertices and let us order the vertices of G starting with the source ones. Let d be its weighted in-degree

vector and L its weighted in-degree Laplacian. Then for any real numbers $c_1, \dots, c_l$ there exist real numbers $x_{l+1}, \dots, x_n$ such that the vector

$$x = (c_1, \dots, c_l, x_{l+1}, \dots, x_n)$$

satisfies

$$L^T x = d. \tag{5}$$

Moreover, let $\mathcal{D}$ be the set of differences of x defined by

$$\mathcal{D} = \{x_j - x_i \,|\, a_{ij} > 0,\ i, j \in G\}.$$

Then the weighted, by edge weights, mean of $\mathcal{D}$ is 1.

Proof. Since G is simply forward influenced with l source vertices, we know from [3] that the dimension of $\ker(L)$ is l. We write the linear system (5) as

$$d_i x_i - \sum_j a_{ji} x_j = d_i.$$

The first l equations correspond to source vertices and become $0 = 0$, so we can choose any value for x_i, $i \in \{1, \dots, l\}$. Moreover, since the dimension of $\ker(L)$ is l, the rest of the equations can be solved. So we conclude that such x exists.

We have

$$\begin{aligned}
\text{Mean}(\mathcal{D}) &= \frac{\sum_i \sum_j a_{ji}(x_i - x_j)}{\sum_i \sum_j a_{ji}} \\
&= \frac{\sum_i (\sum_j a_{ji} x_i - \sum_j a_{ji} x_j)}{\sum_i d_i} \\
&= \frac{\sum_i (d_i x_i - \sum_j a_{ji} x_j)}{\sum_i d_i} \\
&= \frac{\sum_i d_i}{\sum_i d_i} = 1.
\end{aligned}$$

□

Lemma 1.2 is a straightforward corollary.

Lemma 3.2. Let G be a hierarchically decomposable graph, let $\Gamma_1, \dots, \Gamma_l$ be its minimal source (resp. sink) subgraphs and let H be the simply forward (resp. backward) influenced subgraph of G. Let m be the sum of weights of all edges in H and l_i the sum of weights of all edges in Γ_i. Then

$$\eta(G) = \frac{\sum_i \eta(\Gamma_i) l_i}{m + \sum_i l_i},$$

where η denotes the forward (resp. backward) democracy coefficient.

Proof of Lemma 3.2. Let χ_G be the weighted sum of hierarchical differences of graph G, weighted by the edge weights. It is trivially true that

$$\chi_G = \chi_H + \sum_i \chi_{\Gamma_i}.$$

From Lemma 3.1 we know that no matter what are the values of the source vertices of H, the weighted mean of differences will be 1. This means that $\chi_H = m$. Since the weighted mean of forward differences of Γ_i is $1 - \eta_f(\Gamma_i)$, we have $\chi_{\Gamma_i} = (1 - \eta_f(\Gamma_i))l_i$. From this we get

$$\chi_G = \chi_h + \sum_i \chi_{\Gamma_i} = m + \sum_i (1 - \eta_f(\Gamma_i))l_i.$$

Then we have

$$\begin{aligned} \eta_f(G) &= 1 - \frac{\chi_G}{m + \sum_i l_i} \\ &= \frac{m + \sum_i l_i - \chi_G}{m + \sum_i l_i} \\ &= \frac{m + \sum_i l_i - m - \sum_i (1 - \eta_f(\Gamma_i))l_i}{m + \sum_i l_i} \\ &= \frac{\sum_i \eta_f(\Gamma_i) l_i}{m + \sum_i l_i}. \end{aligned}$$

This proves the lemma for the forward democracy coeffient. By doing the same for G^T we prove the lemma for the backward democracy coeffient. □

Based on Lemma 3.2, we conjecture that the democracy coefficient of a graph cannot be arbitrarily small.

Conjecture 3.3. Let G be an unweighted directed graph with m edges. Then

$$\eta_f(G), \eta_b(G) \notin (0, \tfrac{2}{m}) \cup (\tfrac{2}{m}, \tfrac{3}{m}).$$

Moreover, if $m > 3$, then $\eta_f(G) = 2/m$ if and only if G is weakly connected and its minimal source subgraphs are all source vertices except one which is a source pair, i.e. a strongly connected subgraph with 2 vertices.

Lemma 3.4. A minimal source (or sink) subgraph is strongly connected.

Proof. Let G be a source subgraph that is not strongly connected. This means that there exist vertices i and j such that there is no directed path from i to j. We define J to be the set of all vertices from which there is a directed path to j. We define J^c to be the set of all vertices from which there is no directed path to j. By definition $i \in J^c$. By construction there are no directed edges from J^c to J, which implies that J^c is a source subgraph of G, which is a contradiction.

Since a minimal sink subgraph of G is a minimal source subgraph of G^c, we get that minimal sink subgraphs are also strongly connected. □

Lemma 3.5. A weakly connected graph is hierarchically decomposable if and only if it is not strongly connected.

Proof. If the graph is strongly connected, then the only source and sink subgraph is the graph itself, so by definition it is not hierarchically decomposable.

For the other direction, we assume that the graph is not strongly connected and we repeat the construction of J and J^c from the proof of Lemma 3.4. From J^c we can construct Γ_1, a minimal source subgraph of G and since we know that Γ_1 is not the whole G, we get that G is hierarchically decomposable. □

Corollary 3.6. Let G be a weakly connected graph. G is hierarchically decomposable, if and only if G^T is hierarchically decomposable.

Proof. This is a direct corollary of Lemma 3.5, since G is strongly connected if and only if G^T is strongly connected. □

Lemma 3.7. Let G be a strongly connected graph. Then $\ker(L)$ is spanned by a positive integer vector.

Proof. As G is strongly connected, the kernel of L is 1-dimensional, see [3]. Moreover, Proposition 4.1 in [4] shows that there exists a positive integer vector that belongs to $\ker(L)$. These two facts prove the lemma. □

Lemma 3.8. Let G be a hierarchically decomposable directed graph and let $\Gamma_1, \ldots, \Gamma_l$ be its minimal source subgraphs. Let d be its in-degree vector, L be its in-degree Laplacian and L_i be the in-degree Laplacian of Γ_i. Then

1. $\ker(L_i)$ is spanned by a positive vector κ_i.
2. $\ker(L)$ is spanned by the non-negative vectors $k_i = (0, \ldots, 0, \kappa_i, 0, \ldots, 0)$, where $i \in \{i, \ldots, l\}$ and the position of κ_i in k_i corresponds to the position of L_i in L.
3. $k_i d = 0$ if Γ_i is just a single vertex and $k_i d > 0$ otherwise.

Proof.

1. Since Γ_i is a minimal source subgraph, by Lemma 3.4 it is strongly connected and by Lemma 3.7 $\ker(L_i)$ is spanned by a positive vector κ_i.
2. Since there are l minimal source subgraphs the dimension of $\ker(L)$ is l, see [3]. By renaming the vertices, the Laplacian L can be brought to the form

$$L = \begin{pmatrix} L_1 & 0 & \ldots & 0 & C_1 \\ 0 & L_2 & \ldots & 0 & C_2 \\ \vdots & \vdots & \ddots & \vdots & \vdots \\ 0 & 0 & \ldots & L_l & C_l \\ 0 & 0 & \ldots & 0 & C_{l+1} \end{pmatrix}.$$

It is straightforward to check that the vector $k_i = (0, \ldots, 0, \kappa_i, 0, \ldots, 0)$ is in $\ker(L)$. Since we can construct l such vectors and by construction they are orthogonal, they form a basis of $\ker(L)$.

3. Without loss of generality we we will prove this only for Γ_1. If Γ_1 is a single vertex then L_1 is just the 1×1 zero matrix. This means that $k_1 = (1, 0, \ldots, 0)$ and $d = (0, d_2, \ldots, d_n)$, thus $k_1 d = 0$. If Γ_1 is a strongly connected graph with m vertices, then κ_1 is a positive m-vector and the in-degree vector has the form $d = (d_1, \ldots, d_m, \ldots, d_n)$. This means that $d_1, \ldots, d_m > 0$, so we get that $k_1 d > 0$. □

Lemma 3.9. Let G be a weakly connected graph, L be its in-degree Laplacian and d be its in-degree vector. Then a vector x that satisfies $L^T x = d$ exists if and only if G is simply forward influenced.

Proof. Lemma 3.1 states that if a graph is simply forward influenced, then the system $L^T x = d$ can be solved.

For the converse we recall from linear algebra that such x exists if and only if the orthogonal projection of d onto $\ker(L)$ is the 0 vector. Let us assume that there exists a vector x that satsfies $L^T x = d$. Let Γ_1, ..., Γ_l be the minimal source subgraphs of G. Let k_i, where $i \in \{1, \ldots, l\}$, be the vectors that span $\ker(L)$. Since the vector x exists, this means that $dk_i = 0$ for all $i \in \{1, \ldots, l\}$. Then by virtue of Lemma 3.8, Γ_i is a single vertex , thus G is simply forward influenced. □

Lemma 3.10. Let $b := (b_1, \ldots, b_n)$ be the vector, whose entries are defined by

$$b_i := d_i - \left(d_i g_i - \sum_j a_{ji} g_j \right),$$

then b has non-negative entries and is the preojection of d onto the kernel of L.

Proof. By definition of the entries of b and recalling that $M = L^T$ we have

$$b = d - Mg = d - MM^+ d = (I - MM^+)d.$$

The matrix $I - MM^+$ is the orthogonal projector onto the kernel of $M^\mathsf{T} = L$, see [5]. So b is indeed the orthogonal projection of d onto $\ker(L)$. Lemma 3.8 shows that the kernel of L is spanned by non-negative vectors. Since d is also a vector with non-negative entries, the projection of d onto $\ker(L)$ is a vector with non-negative entries. □

Lemma 3.11. Let G be a simple directed graph, let d be its in-degree vector, let L be its in-degree Laplacian and let b be the vector defined in Lemma 3.10. Then

$$\eta_f(G) = \frac{\sum_i b_i}{\sum_i d_i}.$$

Proof. Let g be the vector of forward hierarchical levels of G. From the definition of Section 3.2 we have

$$\eta_f(G) = 1 - \frac{\sum_i \sum_j a_{ji}(g_i - g_j)}{\sum_i \sum_j a_{ji}}.$$

We compare this with the definition of b and we have

$$\begin{aligned}\eta_f(G) &= \frac{\sum_i \sum_j a_{ji} - \sum_i \sum_j a_{ji}(g_i - g_j)}{\sum_i \sum_j a_{ji}} \\ &= \frac{\sum_i (d_i - d_i g_i + \sum_j a_{ji} g_j)}{\sum_i d_i} \\ &= \frac{\sum_i b_i}{\sum_i d_i}.\end{aligned}$$

□

Proof of Lemma 3.4. Since $\eta_f(G) = \eta_b(G^T)$, we will prove the forward version of the lemma only. The backward version of the lemma is proved by repeating the process for G^T.

We know from Lemma 3.11 that $\eta_f(G) = \sum_i b_i / \sum_i d_i$ and since b has non-negative enties, we have that $\sum_i b_i > 0$. This proves the first assertion of the lemma.

The second assertion will be proved in two steps. Let g be the vector of HLs of G. First assume that G is simply forward influenced. This means that the forward hierarchical levels vector g satisfies the equation $Mg = d$, i.e. $d_i g_i - \sum_j a_{ji} g_j = d_i$ for all i. This gives

$$\begin{aligned}\eta_f(G) &= 1 - \frac{\sum_i \sum_j a_{ji}(g_i - g_j)}{\sum_i \sum_j a_{ji}} \\ &= 1 - \frac{\sum_i (d_i g_i - \sum_j a_{ji} g_j)}{\sum_i d_i} \\ &= 1 - \frac{\sum_i d_i}{\sum_i d_i} = 0.\end{aligned}$$

For the other direction we assume that G is a weakly connected graph with $\eta_f(G) = 0$, thus $\sum_i b_i = 0$. This means that $b = 0$. This implies that the projection of d onto the kernel of M^T is 0, so d is in the range of M. From this we deduce that the linear system $Mg = d$ can be solved and we use Lemma 3.9 to deduce that G is simply forward influenced. □

Proof of Lemma 3.5. First we prove that a weakly connected, balanced graph is strongly connected. Let us assume that the graph is not strongly connected and let us separate G into a source subgraph Γ and its complement $G \setminus \Gamma$. We know that there cannot be a directed edge from $G \setminus \Gamma$ to Γ, but there has to be at least one directed edge from Γ to $G \setminus \Gamma$. However, since the sum of in-degrees in Γ equals the sum of out-degrees, this is impossible, so G is strongly connected.

Let L be the in-degree Laplacian of G. Because G is balanced, every row and every column of L sums to 0. From this we deduce that the vector $\mathbb{1} = (1, \dots, 1)$ is in the kernel of both L and L^T. Since G is strongly connected, the kernel of L is 1-dimensional, see [3]. So $\mathbb{1}$ spans both $\ker(L)$ and $\ker(L^\mathsf{T})$.

The projection of d onto $\ker(L)$ is

$$b = \frac{d \cdot \mathbb{1}}{\mathbb{1} \cdot \mathbb{1}} \mathbb{1} = \frac{\sum_i d_i}{n} \mathbb{1}.$$

This means that $\sum_i b_i = \sum_i d_i$. Then by Lemma 3.11 we get $\eta_f(g) = 1$. Since G^T is also balanced, we have $\eta_b(G) = \eta_f(G^T) = 1$. □

Lemma 3.12. Let G be a positively weighted, weakly connected graph, directed graph, let b be the vector defined in Lemma 3.10, let d_i and $\eta_f(G, i)$ be respectively the degree and the forward influence centrality of vertex i. Then if $d_i > 0$, $\eta_f(G, i) = b_i/d_i$.

Proof. Assume that $d_i > 0$. We have

$$\begin{aligned} \eta_f(G, i) &= 1 - \frac{\sum_j a_{ji}(g_i - g_j)}{\sum_j a_{ji}} = 1 - \frac{\sum_j a_{ji} g_i - \sum_j a_{ji} g_j}{d_i} \\ &= 1 - \frac{d_i g_i - \sum_j a_{ji} g_j}{d_i} = 1 - \frac{d_i - b_i}{d_i} = \frac{b_i}{d_i}. \end{aligned}$$

□

Proof of Lemma 3.8. For the forward version of the lemma, we know that if $d_i = 0$ then the vertex i is a source subgraph and by definition $\eta_f(G, i) = 1$. If $d_i > 0$, from Lemma 3.12 we have $\eta_f(G, i) = b_i/d_i$.

Recall that b is the orthogonal projection of d on $\ker(L)$. We use Lemma 3.8 and we see that for any i with $d_i > 0$, $b_i = 0$ if and only if $i \in G \setminus \cup_i \Gamma_i$. The same argument for G^T proves the backward version of the lemma.

For the last part of the lemma, we know from Lemma 3.7 that if the graph is strongly connected, then the $\ker(L)$ is spanned by a positive vector, so for every i, $b_i > 0$.

For the converse, we assume that there exist $i \in G$ such that $\eta_f(G, i) > 0$ and $\eta_b(G, i) > 0$. Since $\eta_f(G, i) > 0$, then there exists a minimal source subgraph Γ such that $i \in \Gamma$. Similarly, since $\eta_b(G, i) > 0$, then there exists a minimal sink subgraph Δ such that $i \in \Delta$. From Lemma 3.4 we know that both Γ and Δ are strongly connected. Moreover, since they have a common vertex, they are actually the same subgraph. This means that since Γ is both a minimal source and minimal sink subgraph, there are no directed edges between Γ and the rest of G. This means that since G is weakly connected, $\Gamma = G$. This concludes the proof. □

Proof of Lemma 1.3. Let b be the vector defined in Lemma 3.10. By definition of b we get

$$d_i g_i - \sum_j a_{ji} g_j = d_i - b_i.$$

If $d_i > 0$ we have $e_i = b_i/d_i$ and we get

$$d_i g_i - \sum_j a_{ji} g_j = d_i(1 - e_i).$$

If $d_i = 0$, then we have $e_i = 1$ and the above relation holds. This means that in matrix form we have $Mg = d \circledast (\mathbf{1} - e)$. □

Proof of Lemma 2.1. Since G is strongly connected, d has positive entries, so D is invertible. Since the graph is strongly connected then from Lemma 3.12 we get that $e_i = b_i/d_i$ or $e = D^{-1}b$ in matrix form, where b is the vector defined in Lemma 3.10.

Let A be the adjacency matrix of G and Π be the transition probability matrix for the random source seeker. Then we have $A^T = D\Pi$.

We define $R = I - \Pi^T$ and from the Fundamental Theorem of Markov Chains it follows that the kernel of R is one dimensional and $Rp = 0$. We have that $RD = (I - \Pi^T)D = D - \Pi^T D = D - A = L$. Since $b \in \ker(L)$, we get $0 = Lb = LDe = RD^2e$. Because the kernel of R is one-dimensional, we get that there exists c such that $cp = D^2e$. We know that p and e are positive vectors, so $c > 0$. Finally we have $|c|/, \|p\|_1 = c = \|D^2e\|_1$. □

Proof of Lemma 3.9. We apply Lemma 2.1 on G^T.

□

4 Graph Generation

4.1 Preferential Preying Model

The *Preferential Preying Model* (PPM) was introduced in [6] as a way to generate graphs that are similar to food webs. In order to generate a graph with PPM we choose N the number of vertices, B the number of source vertices, E the number of edges and T the "temperature". The PPM algorithm is:

1. We introduce B source vertices and no edges.
2. We choose uniformly at random one of the existing vertices i and we add a new vertex j and the edge $i \to j$.
3. We repeat step 2 until we have N vertices in total.
4. We assign each vertex i its trophic level s_i according to the graph we have up to this point.
5. From all possible edges $i \to j$ such that j is not a source vertex, we choose $L - N + B$ edges, each with probability proportional to

$$\mathbb{P}(a_{ij} = 1) \propto \exp\left(-\frac{(s_j - s_i - 1)^2}{2T^2}\right).$$

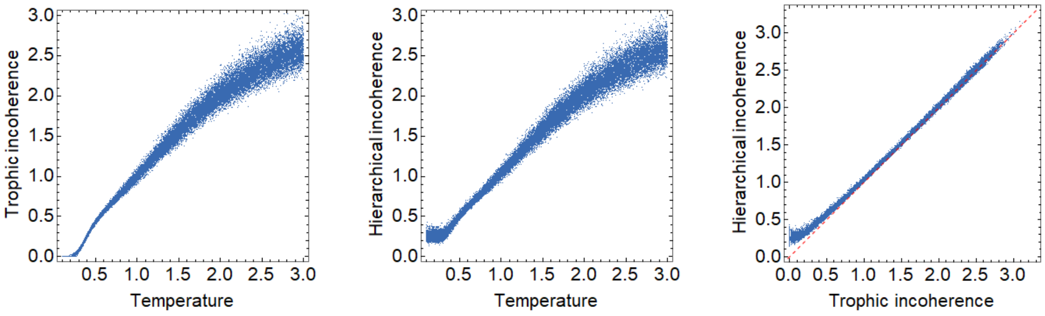

(a) (b) (c)

Figure 3: The correlation between temperature, trophic incoherence and hierarchical incoherence in PPM graphs. (a) Scatter plot of trophic incoherence over temperature. (b) Scatter plot of forward hierarchical incoherence over temperature. (c) Scatter plot of forward hierarchical incoherence over trophic incoherence. Notice that there is some divergence between them only for small values of trophic incoherence.

All PPM graphs are simply influenced graphs, so the democracy coefficient is 0. In Figure 3 we see that the trophic incoherence coefficient almost equals the forward hierarchical incoherence coefficient if they are not small. This is due to the fact that a perfectly layered graph will have 0 trophic incoherence because its source vertices have all the same level. In contrast, the forward hierarchical levels of the source vertices vary depending on the connectivity of the graph. This means that the forward hierarchical incoherence is rarely 0. This can be seen by comparing the graphs in Figures 1a and 2a.

4.2 Non-Source preferential preying model

The *non-source preferential preying model* (NSPPM) algorithm is a modification of the PPM algorithm. In practice we generate a PPM graph and then we make sure that there are no source vertices. We choose N the number of vertices, B the number of source-like vertices, E the number of edges and T the "temperature". The NSPPM algorithm is:

1. We introduce B source vertices and no edges.
2. We choose uniformly at random one of the existing vertices i and we add a new vertex j and the edge $i \to j$.
3. We repeat step 2 until we have N vertices in total.
4. We assign each vertex i its trophic level s_i according to the graph we have up to this point.
5. From all possible edges $i \to j$ such that j is not a source vertex, we choose $L-N+B$

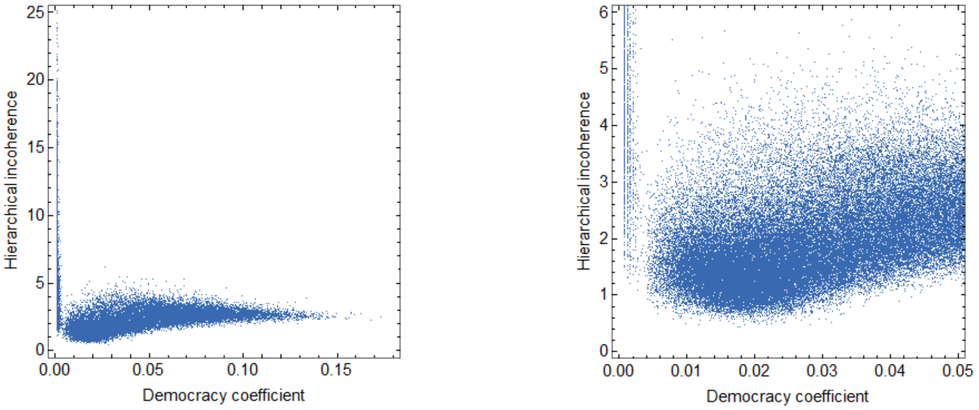

(a) (b)

Figure 4: The scatter plots of hierarchical incoherence over democracy coefficient for NSPPM graphs. Two different regions are visible, one with democracy coefficient between 0 and 20/2500 and hierarchical incoherence that go up to 25 and another band with hierarchical incoherence greater than 20/2500 and hierarchical incoherence between 0.6 and 5. (a)A scatter plot with hierarchical incoherence between 0 and 60. (b) A zoomed in scatter plot with hierarchical incoherence between 0 and 6.

edges, each with probability proportional to

$$\mathbb{P}(a_{ij} = 1) \propto \exp\left(-\frac{(s_j - s_i - 1)^2}{2T^2}\right).$$

6. We pick a source vertex i with in-degree 0, we pick another vertex j with probability proportional to $\exp(-s_j)$ and we add the edge $j \to i$.

7. We repeat step 6 until all source vertices have in-degree 1.

We find that NSPPM graphs can be separated into two types. The ones with very small democracy coefficient (smaller than 20/2500) and the one with a democracy coefficient bigger than 20/2500. We can see in Figures 4 and 5 the two different types produce very different distributions of hierarchical incoherence. The value 20/2500 was chosen empirically based on the results. If Conjecture 3.6 is true, then it would mean that for the graphs in the first category the sum of edges in their source subgraphs is 20 or lower, which we always found to be the case. We found that roughly 2% of the generated graphs fell into this category. This percentage varied a bit with T, with lower T having higher probability of generating this type of graph. We see that graphs of the first type have a very wide range of hierarchical incoherence. This depends on the connectivity of the source subgraph and the topology of the simply forward influenced subgraph. When the simply forward influenced subgraph has no clear/strong hierarchy, i.e. resembles a Erdös-Rényi graph, the hierarchical incoherence of the graph tends to be high. Moreover the hierarchical incoherence tends to be high when the out-neighbours of its source subgraphs are not clustered together.

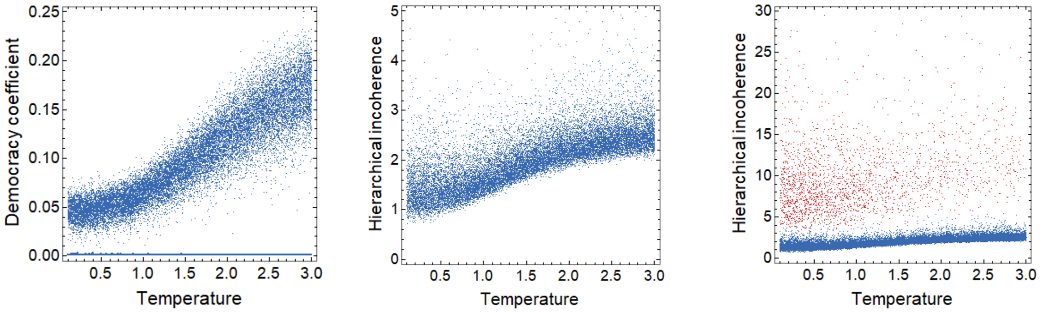

(a) (b) (c)

Figure 5: Scatter plots of hierarchical incoherence and democracy coefficient over temperature for NSPPM graphs. (a) Democracy coefficient over temperature. The graphs with democracy coefficient less or equal than 20/2500 form a very tight band on the bottom of the figure. The graphs with democracy coefficient greater than 20/2500 form a much wider band and there is a clear gap between them. (b) Hierarchical incoherence over temperature of graphs with democracy coefficient greater than 20/2500. A well defined and relatively narrow band can be seen. (c) Hierarchical incoherence over temperature of all graphs. For clarity, graphs with democracy coefficient less or equal to 20/2500 are represented by red points.

5 Contagion Dynamics

For contagion dynamics we used a simple Susceptible-Infected-Susceptible epidemic model [7]. Even though the model is not very realistic, its simplicity makes it relatively popular. Our aim was to show that the hierarchical structure of a graph can give us dynamical insight, so this model was deemed sufficient. We assume that each vertex has two states, *susceptible* and *infected*. Following [8], the probability that vertex i is infected at time $t+1$ is

$$\mathbb{P}(i \text{ is infected at time } t+1) = f_i(t)^{\alpha},$$

where $f_i(t)$ is the fraction of i's in-neighbours which are infected at time t and α is a positive parameter that controls the infection rate. Notice that the probability does not depend on the state of i at all. The parameter α is used to tune how infectious the "disease" is. In our simulation we use α in the range $[0.5, 1.7]$ with step 0.05. A small α means it easy for a vertex to get infected and a large α means it hard for a vertex to get infected. For example when $\alpha = 0.5$, a vertex has probability at least 50% to be infected if at least a quarter of its in-neighbours are infected. On the other hand, when $\alpha = 1.7$, a vertex has probability at least 50% to be infected if at least two thirds of its in-neighbours are infected.

We generated graphs using NSPPM with $N = 500$, $B = 25$ and $E = 2500$ for temperatures in the range $[0.2, 3]$ with step 0.025. We created 1000 graphs for each set of parameters and computed the democracy coefficient and the hierarchical incoherence of each. Then for each values of α we infected the 25 vertices with the lowest hierarchical

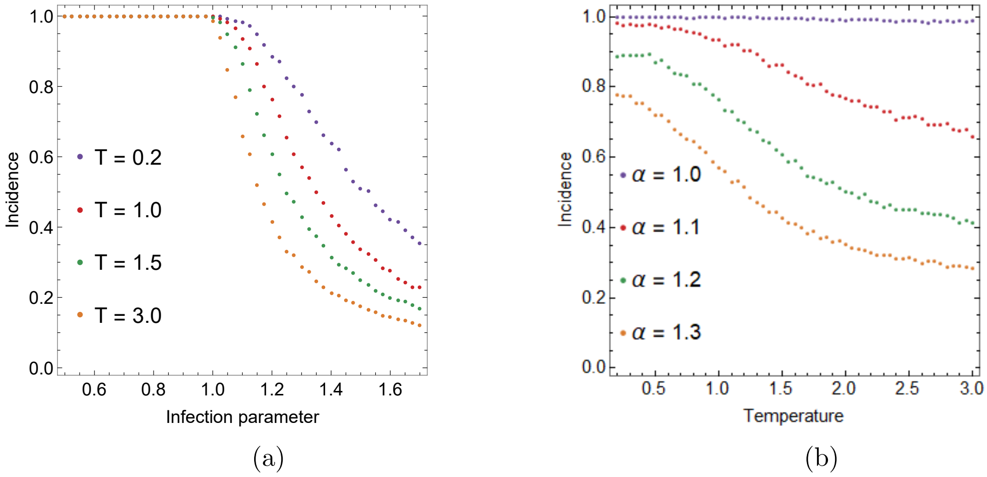

Figure 6: Scatter plot of average incidence values from Monte Carlo simulations of the infection spreading with varying temperature T and infection parameter α. The average is taken over 1000 runs. (a) Incidence against α for different values of T. (b) Incidence against T for different values of α.

level and noted the incidence 1000 times. The simulation continued until the incidence became 1 or no vertex was infected or it reached time step 1000.

We found that on graphs with small democracy coefficient, of the first type, the simulation tended to either end quickly with incidence 1 or time out. This is due to the fact that when a graph has a small forward influencing subgraph, all the vertices in the subgraph tend to have low hierarchical levels. This means that the subgraph starts infected, it stays infected for ever. For small α the infection spreads very quickly everywhere so the simulation exits with incidence 1, but for large α the infection never disappears so the simulation times out. Because of this we have excluded these graphs from the results.

On graphs of the second type the infection behaved differently depending on the hierarchical incoherence. On graphs with high incoherence, the infection spread seemingly randomly. On graphs with low incoherence the infection looked like a wave that travelled through the graph. However since NSPPM graphs are PPM graphs where the source vertices gained an in-neighbour, there can be vertices with low hierarchical level that have only one in-neighbour with relatively high hierarchical level. This means that as the infection wave travels through graph, the in-neighbour becomes infected and since it is the only in-neighbour the vertex become infected. This creates another wave that travels through the graph. So in graphs with democracy coefficient higher than 20/2500 and low hierarchical incoherence, close to 1, we usually found that the infection spread through the graph in periodically generated waves.

Figure 6 contains scatter plots of incidence against temperature and infection parameter. Graphs of the first type were discarded as well as ones in which the simulations timed out. The average was taken out of 1000 non timed out simulations. We see that

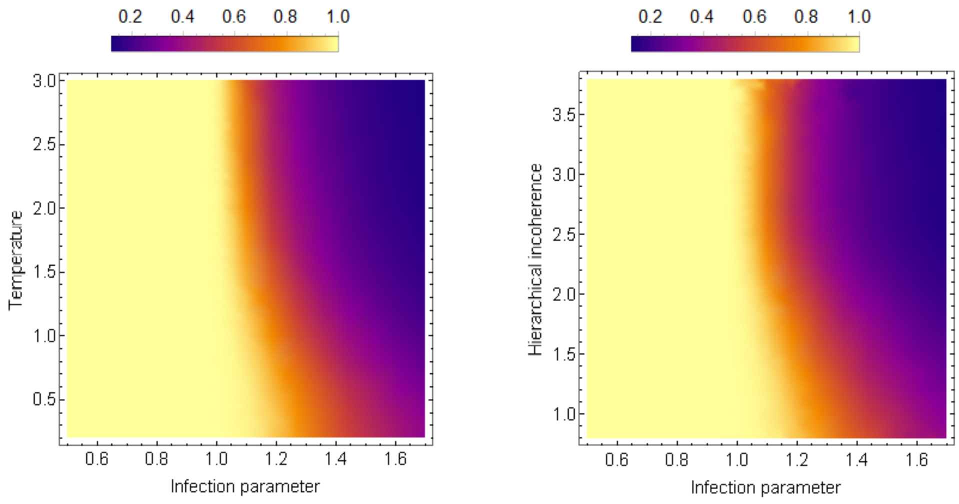

(a) (b)

Figure 7: Heat map of average incidence values from Monte Carlo simulations of the infection spreading. (a) Incidence against α and T. (b) Incidence against α and $\rho_f(G)$.

when α is 1 or smaller the incidence is practically 1. Once α becomes larger than 1, then T starts playing a role. We see that higher temperature means lower incidence. Scatter plots of incidence against incoherence and infection parameter can be seen in Figure 3. Incoherence varied between the interval $(0.8, 3.8)$. Since the incoherence of a graph cannot be chosen, each average was taken over graphs with incoherence in a small interval. Because there were fewer graphs towards the edges of the interval, the error is larger there. This is visible on the graph. As expected higher incoherence leads to lower incidence. Heat maps of incidence can be seen in Figure 7. The first figure is the heat map of incidence against infection parameter and temperature and the second figure is the heat map of incidence against infection parameter and hierarchical incoherence.

6 Time complexity

The calculation of hierarchical levels ends up being a linear convex minimization problem.For such problems we have efficient algorithms. However, we cannot pin down a time complexity for it, as this depends strongly on the sparsity of the graph.

We have compared the time needed to compute the hierarchical structure (HS) of a graph with the time needed to compute the betweenness centrality (BC) and the closeness centrality (CC) of the same graph. NSPPM graphs were used, which were implemented in Julia language using the LightGraphs library. The calculation of the hierarchical structure was done using the Julia language implementation of the algorithm and the closeness and betweenness centralities were computed using functions provided by the LightGraphs library. Note that the HS algorithm computed the forward hierarchical

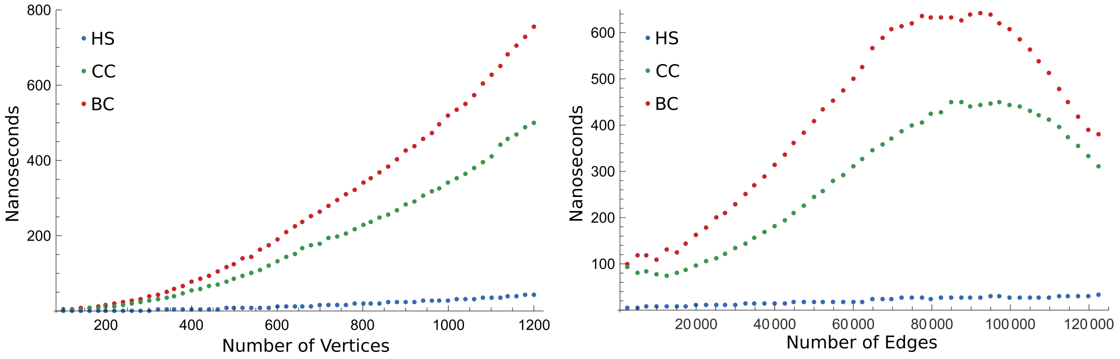

(a) (b)

Figure 8: The figure shows the average time needed to compute three graph metrics, betweenness centrality (BC), closeness centrality (CC) and hierarchical structure (HS). The average is taken over 100 graphs. The time is measured in nanoseconds. Figure (a) shows the time needed when the number of vertices is varied. The number of edges in the graphs was 5% of the edges in a complete graph with the same number of vertices. Figure (b) shows the time needed when the number of edges is varied. The graphs had 500 vertices and the number of edges varied from 2% to 96% of the edges in a complete graph.

levels and the forward influence centralities. The average is taken over 100 graphs. The calculations were performed on a laptop with an Intel i7-8850H processor and 16GB of ram.

The results are presented in Figure 8. Unsurprisingly, all times increase monotonically with the number of vertices. When the graphs had 1200 vertices, the times for HS, CC and BC were 42.16, 501, 23 and 755.78 nanoseconds respectively.

For fixed number of vertices, only HS times increased monotonically with the number of vertices. The maximum time was 32.54 nanoseconds and happened when edges were 96% of those of a complete graph. On the other hands the CC times have a maximum around 75%. This maximum is 450.46 nanoseconds. Similarly, the BC times have a maximum around 70%, that is 642.52 nanoseconds.

References

[1] Raymond L Lindeman. “The trophic-dynamic aspect of ecology”. In: *Ecology* 23.4 (1942), pp. 399–417.

[2] Stephen Levine. “Several measures of trophic structure applicable to complex food webs”. In: *Journal of Theoretical Biology* 83.2 (1980), pp. 195–207.

[3] John S Caughman and JJP Veerman. “Kernels of directed graph Laplacians”. In: *the electronic journal of combinatorics* 13.1 (2006), p. 39.

[4] Anders Björner and László Lovász. “Chip-firing games on directed graphs”. In: *Journal of algebraic combinatorics* 1.4 (1992), pp. 305–328.

[5] Gene H Golub and Charles F Van Loan. “Matrix Computations, Johns Hopkins U”. In: *Math. Sci., Johns Hopkins University Press, Baltimore, MD* (1996).

[6] Samuel Johnson et al. “Trophic coherence determines food-web stability”. In: *Proceedings of the National Academy of Sciences* 111.50 (2014), pp. 17923–17928.

[7] Romualdo Pastor-Satorras et al. “Epidemic processes in complex networks”. In: *Reviews of modern physics* 87.3 (2015), p. 925.

[8] Janis Klaise and Samuel Johnson. “From neurons to epidemics: How trophic coherence affects spreading processes”. In: *Chaos: An Interdisciplinary Journal of Nonlinear Science* 26.6 (2016), p. 065310.
